# Stability Studies of Thiocolchicoside in Bulk and Capsules Using RP-HPTLC/Densitometry

**DOI:** 10.1155/2013/142628

**Published:** 2013-09-11

**Authors:** Dnyansing K. Rajput, Atul A. Shirkhedkar, Jyoti K. Rajput, Harun M. Patel, Sanjay J. Surana

**Affiliations:** ^1^Department of Quality Assurance, R. C. Patel Institute of Pharmaceutical Education and Research, Shirpur, Dhule 425 405, India; ^2^Department of Pharmaceutical Chemistry, R. C. Patel Institute of Pharmaceutical Education and Research, Shirpur, Dhule 425 405, India; ^3^R. C. Patel Institute of Pharmaceutical Education and Research, Shirpur, Dhule 425 405, India

## Abstract

A new stability-indicating reversed-phase high-performance thin-layer chromatographic (RP-HPTLC) method for densitometric analysis of thiocolchicoside was developed and validated. The chromatograms were developed using aluminum plates pre-coated with silica gel 60 RP-18 F_254_S as a stationary phase and methanol : water (70 : 30 v/v) as a mobile phase. The compact band for thiocolchicoside was observed at *R*_*f*_ value of 0.60 ± 0.02 at an absorption wavelength of 377 nm. The linear regression data for the calibration plots (*r* ^2^ = 0.9984) was found with respect to peak area in the concentration range of 100–600 ng per band. The limit of detection (LOD) and limit of quantification (LOQ) were 9.77 ng and 29.63 ng, respectively. The drug was exposed to acidic and alkaline hydrolysis, oxidation, photo degradation, and dry heat conditions. The peaks of degradation products were well-resolved from the peak of the standard drug with significantly different *R*_*f*_ values. Statistical analysis proved that the established RP-HPTLC method is reproducible, selective, and accurate for the determination of thiocolchicoside in its formulations. The method can effectively separate the drug from its degradation products, and it can be considered as stability-indicating assay.

## 1. Introduction

**Figure 1:**
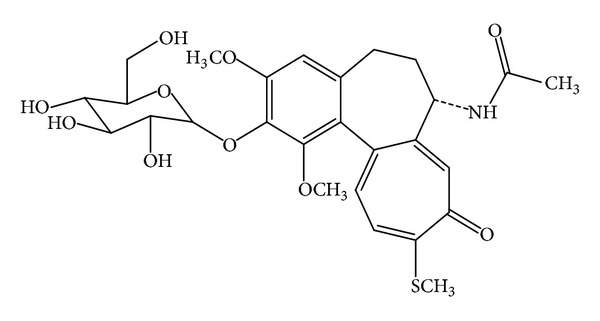
Chemical structure of thiocolchicoside.

Thiocolchicoside is chemically 2-demethoxy-2-glucosidoxythicolchicine (Figure 1) [1]. Thiocolchicoside is a semisynthetic sulfur derivative of colchicoside, a naturally occurring glucoside present in the plant *Gloriosa superba.* Clinically, thiocolchicoside is used for muscle relaxant, anti-inflammatory, and analgesic properties [2]. Few LC-MS-MS methods have been established for the assessment of bioequivalence of thiocolchicoside as a single component [3] and in fixed-dose combination tablet with lornoxicam [4].

Some analytical methods, such as LC-ESI-MS [5] RP-HPLC [6, 7] and UV-Spectrophotometric [8, 9] have been established for the determination of thiocolchicoside alone in bulk and pharmaceutical formulations. 

Thiocolchicoside is available in combination with many other drugs; therefore, several methods such as UV-Spectrophotometric [10–13], RP-HPLC [14–18], and HPTLC [19–23] methods have been studied for the determination of thiocolchicoside in combined dosage forms. 

The International Conference on Harmonization (ICH) guidelines entitled “Stability Testing of New Drug Substances and Products” requires that stress testing can be carried out to elucidate the inherent stability characteristics of the active substance. An ideal stability-indicating method is that which resolves the standard drug as well as its degradation products [24]. Hence, a reliable and rapid determination method needs to be developed, which could also be used to obtain the optimum separation of the degradation components from the parent compound. However, to our knowledge, no article related to the stability-indicating RP-HPTLC determination of thiocolchicoside has ever been mentioned in literature. The objective of the work described in this paper is to establish conditions for identification and quantitative analysis of thiocolchicoside in the presence of its degradation products for assessment of purity of bulk drug and stability of its dosage forms. The suitability of stability-indicating RP-HPTLC method for quantitative determination of thiocolchicoside was proved by validation in accordance with the requirement of ICH guidelines [25].

## 2. Experimental

### 2.1. Chemicals and Reagents

Thiocolchicoside was obtained as a gift sample from Ajanta Pharma. Ltd, Mumbai, India. HPLC grade methanol, HCl, NaOH, and H_2_O_2_ were purchased from Merck Chemicals, India.

### 2.2. HPTLC Instrumentation

The drug standard and samples were spotted in the form of bands of 6 mm width with a CAMAG Linomat microlitre syringe (100 *μ*L, Hamilton, Bunaduz, Switzerland) using a CAMAG Linomat 5-sample applicator (CAMAG Muttenz, Switzerland) with a constant rate of application, 150 nL per second. The plates were prewashed with methanol and activated at 100°C for 10 min prior to chromatography. Chromatography was performed on aluminum plates precoated with silica gel 60 RP-18 F_254_S (20 × 10 cm, E. Merck, Germany). Linear ascending development with methanol: water (70 : 30 v/v) as mobile phase was performed in a 20 × 10 cm twin-trough glass chamber (CAMAG Muttenz, Switzerland). The optimized chamber saturation time for the mobile phase was for 30 min at room temperature (28 ± 2°C). The length of the chromatogram run was approximately 80 mm. Development time of the plate was 25 min. After development, the plates were dried in current of air by an air dryer. Detection of spot was then performed at 377 nm with a CAMAG TLC Scanner 3 in absorbance mode operated by winCATS software version 1.3.0. The source of radiation was a deuterium lamp. Slit dimensions were 6 mm × 0.45 mm, and the scanning speed, 20 mm per second.

### 2.3. Preparation of Stock Standard

Stock standard solution of 1 mg/mL of thiocolchicoside in methanol.

### 2.4. Linearity Study

Stock standard solution in the range of 0.2 to 1.2 mL was transferred into six separate 10 mL volumetric flasks, and volume was made up with methanol. From each of the above solutions, 5 *μ*L was applied on RP-HPTLC plates to obtain concentration in the range of 100 to 600 ng per band. The calibration plot for the method was constructed as peak area versus drug concentration.

### 2.5. Preparation of Sample Solution

To determine the content of thiocolchicoside in capsule, twenty capsules (MYORIL, label claim: 8 mg of thiocolchicoside per capsule) were weighed; the content of capsules was removed; and the average weight was determined. An amount equivalent to 8 mg of thiocolchicoside was transferred to 100 mL volumetric flask containing 50 mL methanol and sonicated for 10 min; volume was adjusted to mark and filtered using Whatmann No. 41 filter paper. A volume of 5 mL was diluted to 10 mL with methanol; resulting solution 5 *μ*L applied on RP-HPTLC plate for assay of thiocolchicoside. The plates were developed and scanned as described above.

### 2.6. Method Validation

The method was validated for following parameters as per ICH guidelines. 

#### 2.6.1. Precision

Repeatability of sample application and measurement of peak area were performed using six replicates of the test concentration (400 ng per band of thiocolchicoside). The intra- and inter-day variation for the estimation of thiocolchicoside was carried out using three replicates at three different concentration levels (200, 300, and 500 ng per band).

#### 2.6.2. Limit of Detection (LOD) and Limit of Quantification (LOQ)

In order to determine detection and quantification limit, thiocolchicoside concentrations in the lower part of the linear range of the calibration curve were used. From the stock standard solution, thiocolchicoside 100, 120, 140, 160, 180, and 200 ng per band was applied in triplicate on RP-HPTLC plate and LOD and LOQ were calculated using the following equations:

(1) $$\begin{array}{c} \text{LOD}=3.3\times \frac{N}{B},\\ \text{LOQ}=10\times \frac{N}{B}, \end{array}$$

where “*N*” is standard deviation of the peak areas of the drugs (*n* = 3), taken as a measure of noise and “*B*” is the slope of the corresponding calibration curve.

#### 2.6.3. Specificity

The specificity of the method was checked by analyzing drug standard and sample. The band for thiocolchicoside in sample was confirmed by comparing the *R*_*f*_ values and spectra of the band with those of drug standard. The peak-purity of thiocolchicoside was confirmed by comparing the spectra at three different levels, that is, peak-start (*S*), peak-apex (*M*), and peak-end (*E*) positions of the band.

#### 2.6.4. Ruggedness

Ruggedness of the method was performed by analyzing 400 ng of thiocolchicoside by two different analysts keeping the same experimental and environmental conditions.

#### 2.6.5. Accuracy

Nine bands of thiocolchicoside sample solution (200 ng per band) were applied on plate, and then the known amount of thiocolchicoside was applied in triplicate at 80, 100, and 120% (160, 200 and 240 ng per band) of the sample concentration (200 ng per band) and reanalysed by the proposed method. This was performed to evaluate the recovery study of the drug at different levels in the formulations. 

#### 2.6.6. Robustness

By making small modifications the mobile phase composition, amount of mobile phase, time from application to development, and time from development to scanning the effects on the results were examined. Mobile phases having different compositions of methanol: water (72 : 28 v/v) and methanol: water (68 : 32 v/v) were tried and chromatograms were run. The plates were prewashed by methanol and activated at 80 ± 5°C for 2, 5, and 8 min prior to chromatography. The robustness of method was performed using six replicates of the same spot (400 ng per band of thiocolchicoside).

### 2.7. Forced Degradation of Thiocolchicoside

#### 2.7.1. Acid and Base Hydrolysis

Accurately weighed quantity 10 mg of thiocolochicoside was separately dissolved in 10 mL methanolic solution of 1.0 M HCl and 0.5 M NaOH, respectively and refluxed for 30 min at 60°C in dark to avoid likely degradative effect of light. A volume of 1.0 mL from the above solutions was separately taken, neutralized and diluted up to 10 mL with methanol. The resultant solutions were applied on the RP-HPTLC plates in triplicates (5 *μ*L each, i.e., 500 ng per band). The chromatograms were developed and scanned as described above.

#### 2.7.2. Oxidative Degradation

For oxidative degradation, accurately weighed quantity 10 mg of thioclochicoside was separately dissolved in 10 mL methanolic solution of 1% v/v H_2_O_2_ and 3% v/v H_2_O_2_, respectively and kept in dark at room temperature for 30 min. After 30 min, 1.0 mL from each of the above solutions were taken and diluted up to 10 mL with methanol. The resultant solutions were applied on RP-HPTLC plates in triplicate (5 *μ*L each, i.e., 500 ng per band). The chromatograms were developed and scanned as described above.

#### 2.7.3. Dry Heat Degradation

Accurately weighed quantity 10 mg of thiocolchicoside was stored at 70°C for 8 h in an oven. It was transferred to 10 mL volumetric flask containing methanol and volume was made up to the mark. The 1.0 mL of above solution was taken and diluted up to 10 mL with methanol. The resultant solution was applied on RP-HPTLC plate in triplicate (5 *μ*L each, i.e., 500 ng per band). The chromatogram was developed and scanned as described above.

#### 2.7.4. Photo Degradation

Accurately weighed quantity 10 mg of thiocolchicoside was dissolved in 10 mL methanol and solutions was kept for period of 24 h in light. An appropriate volume 1.0 mL of above solution was taken and diluted up to 10 mL with methanol. The resultant solution was applied on RP-HPTLC plate in triplicate (5 *μ*L each, i.e., 500 ng per band). The chromatogram was developed and scanned as described above.

## 3. Results and Discussion

### 3.1. Development of Optimum Mobile Phase

**Figure 2:**
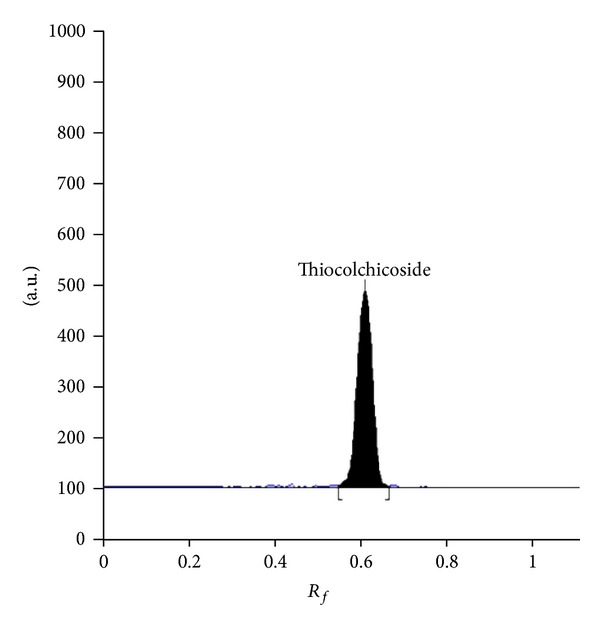
Chromatogram of thiocolchicoside standard with (*R*_*f*_ 0.60 ± 0.02) at 377 nm in methanol: water (70 : 30 v/v) as mobile phase.

For the selection of appropriate mobile phase for the separation of thiocolchicoside, several runs were exercised using mobile phases containing solvents of varying polarities, at different concentration levels. Among the different mobile phase combinations employed, the mobile phase consisting of methanol: water (70 : 30 v/v) gave a sharp and well-defined peak at *R*_*f*_ value of 0.60 ± 0.02 (Figure 2). The well-distinct bands were found when the chamber was saturated with the mobile phase for 30 min at room temperature.

### 3.2. Calibration Curve

**Figure 3:**
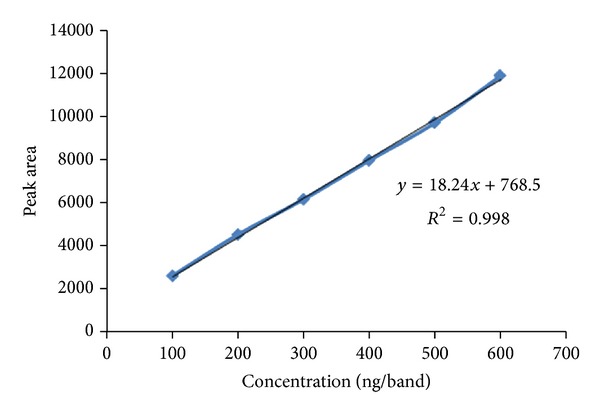
Calibration curve of thiocolchicoside (100–600 ng per band).

The linear regression data for the calibration curves (*n* = 5) showed good linear relationship over the concentration range of 100–600 ng per band. Linear regression equation was found to be *Y* = 18.24*X* + 768.5, *r* ^2^ = 0.9984 (Figure 3).

### 3.3. Validation of Method

The developed method was validated as per ICH guidelines. 

The precision of the method was revealed in terms of % relative standard deviation (% RSD) of the peak area. The results (Table 1) epitomized sounds precision of the method which were determined from the slope of the lowest part of the calibration plot. The LOD and LOQ were found to be 9.77 ng and 29.63 ng, respectively, which indicates the sensitivity of the method, is adequate.

**Figure 4:**
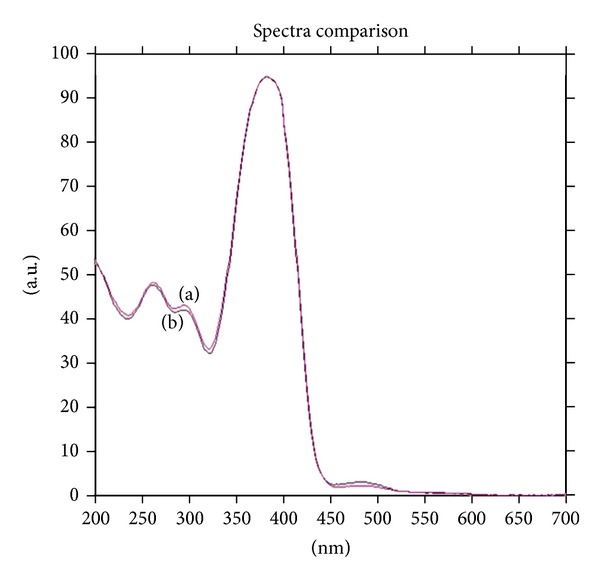
Peak-purity spectra of thiocolchicoside standard (a) and thiocolchicoside extracted from capsule (b) scanned at peak-start, peak-apex and peak-end positions.

The recovery studies were executed out at 80%, 100%, and 120% of the test concentration as per ICH guidelines. The % recovery of thiocolchicoside at all the three levels was found to in the range of 99.92–100.04%. The amounts of drug added and determined and the % recovery are shown in (Table 2). The peak-purity of thiocolchicoside was confirmed by evaluating the spectra studies at peak-start, peak-apex and peak-end positions of the band, that is, *r* ^2^ (*S*, *M*) = 0.9995 and *r* ^2^ (*M*, *E*) = 0.9986 showing specificity of the method (Figure 4). Good correlation (*r* ^2^ = 0.9989) was obtained between drug standard and drug extracted from capsule formulation. The robustness of the method was experimented by making purposeful alteration in the chromatographic conditions, and the effect on the chromatogram was observed. The standard deviation of peak areas was calculated for each parameter, and % RSD was found to be less than 2%. The low values of % RSD values indicate robustness of the method; results are shown in (Table 3). The ruggedness of the method was verified by different analyst and the %RSD was found to be 0.57 and 0.58 it indicate that the method is rugged.

### 3.4. Analysis of the Marketed Formulation

Thiocolchicoside when extracted from capsule formulation demonstrated a single spot having *R*_*f*_ = 0.60 ± 0.02 in the chromatogram. The mean % drug content was found to be 100.27% of the label claim with 0.88% RSD.

### 3.5. Stability-Indicating Property

#### 3.5.1. Acidic Degradation

**Figure 5:**
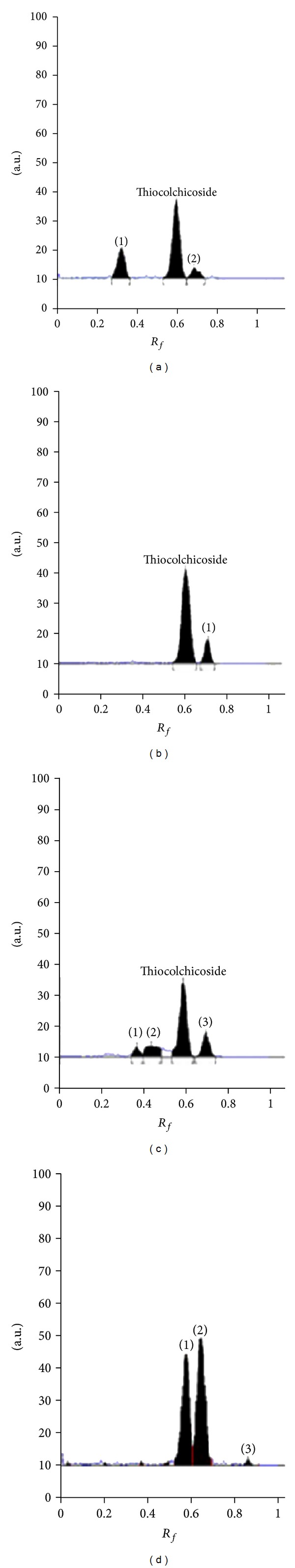
RP-HPTLC chromatograms obtained from thiocolchicoside degraded by (a) acidic hydrolysis (1 M HCl), (b) alkaline hydrolysis (0.5 M NaOH), (c) oxidative stress (1% v/v H_2_O_2_), and (d) oxidative stress (3% v/v H_2_O_2_).

Forced degradation of thiocolchicoside in 1.0 M HCl (60°C for 30 min) was found to be instable and showed two additional peaks at *R*_*f*_ values 0.33 and 0.71 (Figure 5(a)). The spots of the degraded products were well separated from the spot of thiocolchicoside.

#### 3.5.2. Basic Degradation

Thiocolchicoside was found to be instable during alkali hydrolysis in 0.5 M NaOH at 60°C for 30 min. The drug showed one additional peak at *R*_*f*_ value 0.72 with thiocolchicoside remained at *R*_*f*_ 0.60 (Figure 5(b)). The spots of the degraded products were well separated from the drug spots. 

#### 3.5.3. Oxidative Degradation

**Figure 6:**
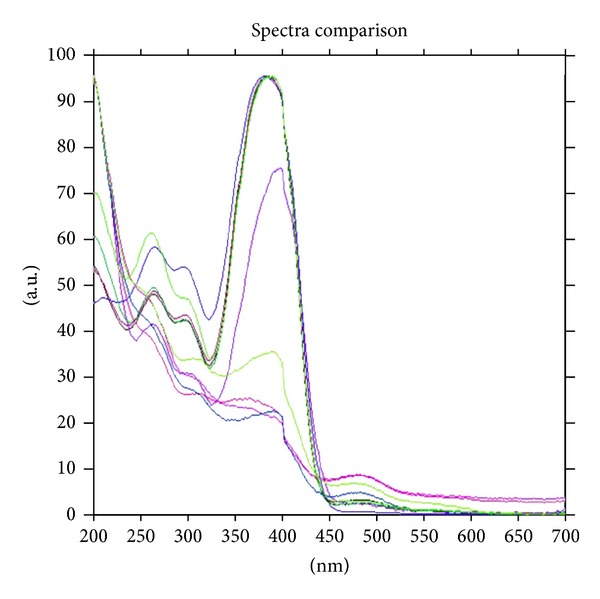
Peak-purity spectra of thiocolchicoside recovered after degradation in 1 M HCl, 0.5 M NaOH, 1% v/v H_2_O_2_, degradants and thiocolchicoside standard scanned at peak-start, peak-apex and peak-end positions.

Thiocolchicoside possesses sulphur atom, which is more susceptible to oxidation by H_2_O_2_. After treatment of thiocolchicoside with 1% v/v H_2_O_2_, three additional peaks at *R*_*f*_ values 0.38, 0.46, and 0.70 were observed along with thiocolchicoside remained at *R*_*f*_ 0.60 (Figure 5(c)). In oxidative degradation with 3% v/v H_2_O_2_ thiocolchicoside underwent complete degradation resulting into two major peaks at *R*_*f*_ values 0.58 and 0.64 and one peak at 0.70, respectively (Figure 5(d)). The peak-purity spectra of thiocolchicoside recovered after degradation in 1 M HCl, 0.5 M NaOH, and 1% v/v H_2_O_2_ and thiocolchicoside standard scanned at peak-start, peak-apex, and peak end positions of the spot are as shown in (Figure 6). The results from the stress testing studies revealed that the method was highly specific for thiocolchicoside. The degradation products were entirely noticeable from the parent compound. No decomposition was identified on exposure of drug solution to sunlight during photo and thermal degradation indicating stability of drug to both conditions. The results of the forced degradation study of thiocolchicoside are summarized in (Table 4).

## 4. Conclusion 

In the present study, forced degradation of thiocolchicoside was performed to elucidate its inherent chemical stability. For this purpose an RP-HPTLC method has been developed. The developed method was found to be simple, rapid, selective, sensitive, and suitable for determination of thiocolchicoside in bulk material and capsule formulation. During the study, it was found that thiocolchicoside is susceptible to acid and base hydrolysis as well as oxidation. As the method is stability-indicating one, it can be used to determine the purity of the drug available from various sources by detecting the related impurities. Besides, it can be concluded that the impurities present in the drug could be due to hydrolysis or oxidation during the processing and storage of the drug. 

## Figures and Tables

**Table 1:** Repeatability and intraday and interday precisions.

Parameters	Concentration (ng per band)	% Amount found	% RSD
Repeatability (*n* = 6)	400	99.39	0.61

Intraday (*n* = 3)	200	100.73	0.64
	300	99.83	0.78
	500	99.18	0.32

Interday (*n* = 3)	200	100.80	1.43
	300	99.65	0.70
	500	99.91	0.50

*n*: number of determinations.

**Table 2:** Recovery studies.

Drug	Initial amount (ng per band)	Amount of drug standard added (%)	% Drug recovered (*n* = 3)	% R.S.D. (*n* = 3)
Thiocolchicoside	200	80	99.92	0.74
		100	100.04	0.88
		120	99.97	0.46

*n*: number of determinations.

**Table 3:** Robustness of the method.

Parameter	± S.D. of peak area (*n* = 6)	% R.S.D. (*n* = 6)
Mobile phase composition (±2 mL)	32.38	0.73
Amount of Mobile phase (±5%)	37.31	0.84
Time from application to development (±10 min)	24.72	0.55
Time from development to scanning (±10 min)	36.77	0.82
Activation of TLC plates	34.40	0.77

*n*: number of determinations.

**Table 4:** Forced degradation studies.

Stress conditions	Temperature	% Recovery (*R*_*f*_)			
		Thiocolchicoside	peak-1	peak-2	peak-3
1.0 M HCl	60°C	78.00 (0.60)	8.22 (0.33)	13.78 (0.71)	
0.5 M NaOH	60°C	85 (0.60)	15 (0.72)	—	—
1% *v/v *H_2_O_2_	RT	67.50 (0.60)	2.18 (0.38)	10.20 (0.46)	20.12 (0.70)
Photodegradation-day light (8 h/day)	RT	92.94 (0.60)	—	—	—
Dry heat	70°C	98.12 (0.60)	—	—	—

RT: Room Temperature.

## References

[1] Ministry of Health & Family Welfare (2010). Indian Pharmacopoeia. *Indian Pharmacopoeial Convention*.

[2] Carta M, Murru L, Botta P (2006). The muscle relaxant thiocolchicoside is an antagonist of GABAA receptor function in the central nervous system. *Neuropharmacology*.

[3] Sutherland FCW, Smit MJ, Herbst L (2002). Highly specific and sensitive liquid chromatography-tandem mass spectrometry method for the determination of 3-desmethylthiocolchicine in human plasma as analyte for the assessment of bioequivalence after oral administration of thiocolchicoside. *Journal of Chromatography A*.

[4] Agarwal S, Das A, Chowhury HR, Sarkar AK, Chattaraj TK, Pal TK (2011). Bioequivalence study of fixed dose combination tablet containing lornoxicam and thiocolchicoside in healthy subjects. *International Journal of Pharmaceutical Sciences Review*.

[5] Erika DG, Silvio A, Giorgio G (2012). Forced degradation study of thiocolchicoside: characterization of its degradation products. *Journal of Pharmaceutical and Biomedical Analysis*.

[6] Umarkar AR, Rewatkar NS, Chaple DR, Thote LT, Chaudhari SB, Bhurat MR (2011). Stability indicating RP-HPLC method for estimation of Thiocolchicoside in capsule dosage forms. *Research Journal of Pharmaceutical, Biological and Chemical Sciences*.

[7] Joshi RR, Gupta KR, Jinnawar KS, Wadodkar SG (2012). Development and validation of stability-indicating RP-HPLC and assay method for determination of thiocolchicoside in capsule. *American Journal of PharmTech Research*.

[8] Acharjya SK, Mallick P, Panda P, Annapurna MM (2010). Spectrophotometric methods for the determination of thiocolchicoside in bulk and pharmaceutical dosage forms. *Journal of Pharmaceutical Education and Research*.

[9] Joshi RR, Gupta KR (2010). UV-Spectrophotometric determination of thiocolchicoside in capsule. *Der Pharma Chemica*.

[10] Umarkar AR, Rewatkar NS, Charde MS, Charde RM (2011). Simultaneous estimation of thiocolchicoside and diclofenac potassium by UV spectrophotometer using multicomponent method. *International Journal of ChemTech Research*.

[11] Acharjya SK, Rajesh Y, Panda P, Mallick P, Annapurna MM (2010). Spectrophotometric methods for simultaneous estimation of etoricoxib and thiocolchicoside in bulk and combined pharmaceutical dosage form. *Journal of Pharmaceutical Education and Research*.

[12] Chaudhari BG, Trivedi JB (2012). Simultaneous spectrophotometric estimation of thiocolchicoside and dexketoprofen trometamol in pharmaceutical dosage form. *International Journal of Biomedical and Advance Research*.

[13] Joshi RR, Gupta KR (2010). Simultaneous UV-Spectrophotometric determination of thiocolchicoside and diclofenac in pharmaceutical formulation. *Der Pharmacia Sinica*.

[14] Umarkar AR, Rewatkar NS, Charde MS, Kasture AV (2011). RP-HPLC method development and validation for estimation of thiocolchicoside and diclofenac potassium in bulk and capsule dosage forms. *Journal of Pharmacy Research*.

[15] Kumar S, Joshi A, Thakur RS, Pathak AK, Shah K (2011). Simultaneous estimation of etoricoxib and thiocolchicoside by RP-HPLC method in combined dosage forms. *Acta Poloniae Pharmaceutica*.

[16] Walash M, Belal F, Eid M, El Abass SA (2011). Simultaneous HPLC determination of thiocolchicoside and glafenine as well as thiocolchicoside and floctafenine in their combined dosage forms. *Journal of Chromatographic Science*.

[17] Dhaneshwar SR, Raut KO, Bhusari VK (2011). Validated HPLC method for simultaneous estimation of paracetamol, aceclofenac and thiocolchicoside in bulk drug and formulation. *Research Journal of Pharmaceutical, Biological and Chemical Sciences*.

[18] Bhavsar SM, Patel DM, Khandhar AP, Patel CN (2010). Validated RP-HPLC method for simultaneous estimation of lornoxicam and thiocolchicoside in solid dosage form. *Journal of Chemical and Pharmaceutical Research*.

[19] Syal P, Sahoo M, Raut R (2012). Development and validation of an HPTLC method for simultaneous estimation of thiocolchicoside and aceclofenac in combined dosage form. *Journal of Planar Chromatography*.

[20] Sahoo M, Syal P, Hable AA, Raut RP, Choudhari VP, Kuchekar BS (2011). Development and validation of HPTLC method for simultaneous estimation of lornoxicam and thiocolchicoside in combined dosage form. *Pharmaceutical Methods*.

[21] Patil ST, Bhusari VK, Dhaneshwar SR (2011). Validated HPTLC method for simultaneous estimation of thiocolchicoside and aceclofenac in bulk drug and formulation. *International Journal of Pharma and Bio Sciences*.

[22] Rajmane VS, Gandhi SV, Patil UP, Sengar MR (2010). High-performance thin-layer chromatographic determination of etoricoxib and thiocolchicoside in combined tablet dosage form. *Journal of AOAC International*.

[23] Gandhi S, Deshpande P, Sengar M (2010). High-performance thin-layer chromatographic determination of diclofenac sodium and thiocolchicoside in fixed dose combination. *International Research Journal of Pharmacy*.

[24] ICH Q1A(R2) stability testing of new drug substances and products.

[25] ICH (2005). *Q2(R1) Validation of Analytical Procedures: Text and Methodology, ICH Harmonized Tripartite Guideline*.

